# PD85 The Role Of Knowledge Translation In The Decision-Making Process For Incorporating Health Technologies In Brazil

**DOI:** 10.1017/S0266462325102122

**Published:** 2025-12-29

**Authors:** Laís da Silva Barbosa, Rafaella Maria Vasconcelos da Nóbrega, Barbara Pozzi Ottavio, Thâmmara Lariane Henriques Tito, Maria Stella Martins Silva D’agostini, Marcela Medeiros de Freitas, Luciene Fontes Schluckebier Bonan

## Abstract

**Introduction:**

Persistent challenges in understanding and applying scientific knowledge also impact decision-makers, hampering the implementation of evidence-based actions. Strategies such as knowledge translation (KT) offer tools for effectively comprehending scientific evidence and incorporating it into decision-making. KT is used by Brazil’s National Committee for Health Technology Incorporation (CONITEC) to adapt scientific evidence for different audiences to help involve relevant stakeholders throughout the HTA process.

**Methods:**

This study aimed to explore how CONITEC applies KT at various stages of its HTA process by listing the main materials produced and discussing their impact. Research was carried out using CONITEC’s website to identify relevant documents produced for different audiences as inputs both for decision-making and social participation. Official documents, public consultations, technical reports, and instruments such as surveys and qualitative analyses were evaluated.

**Results:**

Evidence synthesis is the core of CONITEC’s work through its HTA reports. Reports published by CONITEC include an executive summary and are adapted and translated into a plain language “Report for Society” to make their technical content more accessible. To simplify the assessment of public consultation responses, they are divided into: (i) opinion and experience, mostly sent by associations, patients, and caregivers; or (ii) technical or scientific, which discuss technical aspects of the technology. Responses are analyzed, synthetized, and presented to CONITEC. For opinion and experience, there is often a qualitative synthesis to grasp and translate the most relevant aspects for society.

**Conclusions:**

CONITEC employes several tools to improve the understanding of evidence and amplify its dissemination, which fosters the involvement of diverse stakeholders and increases transparency. Such approaches contribute to: (i) disseminating scientific evidence to diverse audiences and reducing informational inequalities; (ii) disseminating the perspectives shared by stakeholders at CONITEC’s meetings and during public consultation; and (iii) engaging stakeholders, notably decision-makers and patients.

